# Supplementary material for: Investigation of Radiation Effect on Structural and Optical Properties of GaAs under High-Energy Electron Irradiation
Source: Materials (Basel). 2022 Aug 26;15(17):5897. doi: 10.3390/ma15175897 (PMC9457441; doi:10.3390/ma15175897)
Supplement: Supplementary file 1 [file materials-15-05897-s001.zip › materials-1872619-supplementary.pdf]

Authit Phakkhawan [1], Aparporn Sakulkalavek [1], Siritorn Buranurak [2], Pawinee Klangtakai [2,3,4,*], Karnwalee Pangza [5], Nongnuch Jangsawang [5], Sawinee Nasompag [6], Mati Horprathum [7], Suphakan Kijamnajsuk [8] and Sakuntam Sanorpim [9]

[1] Department of Physics, Faculty of Science, King Mongkut's Institute of Technology Ladkrabang, Bangkok 10520, Thailand
[2] Department of Physics, Faculty of Science, Khon Kaen University, Khon Kaen 40002, Thailand
[3] Institute of Nanomaterials Research and Innovation for Energy (IN-RIE), Khon Kaen University, Khon Kaen 40002, Thailand
[4] Thailand Center of Excellence in Physics, Chiang Mai University, P.O. Box 70, Chiang Mai 50202, Thailand
[5] Gems Irradiation Center, Thailand Institute of Nuclear Technology, Nakhon-Nayok 26120, Thailand
[6] Research Instrument Center, Khon Kaen University, Khon Kaen 40002, Thailand
[7] National Electronics and Computer Technology Center (NECTEC), National Science and Technology Development Agency (NSTDA), Khlong Luang 12120, Thailand
[8] National Metal and Materials Technology Center (MTEC), National Science and Technology Development Agency (NSTDA), Khlong Luang 12120, Thailand; suphakk@mtec.or.th
[9] Department of Physics, Faculty of Science, Chulalongkorn University, Phayathai Rd., Patumwan, Bangkok 10330, Thailand
* Correspondence: pawinee@kku.ac.th

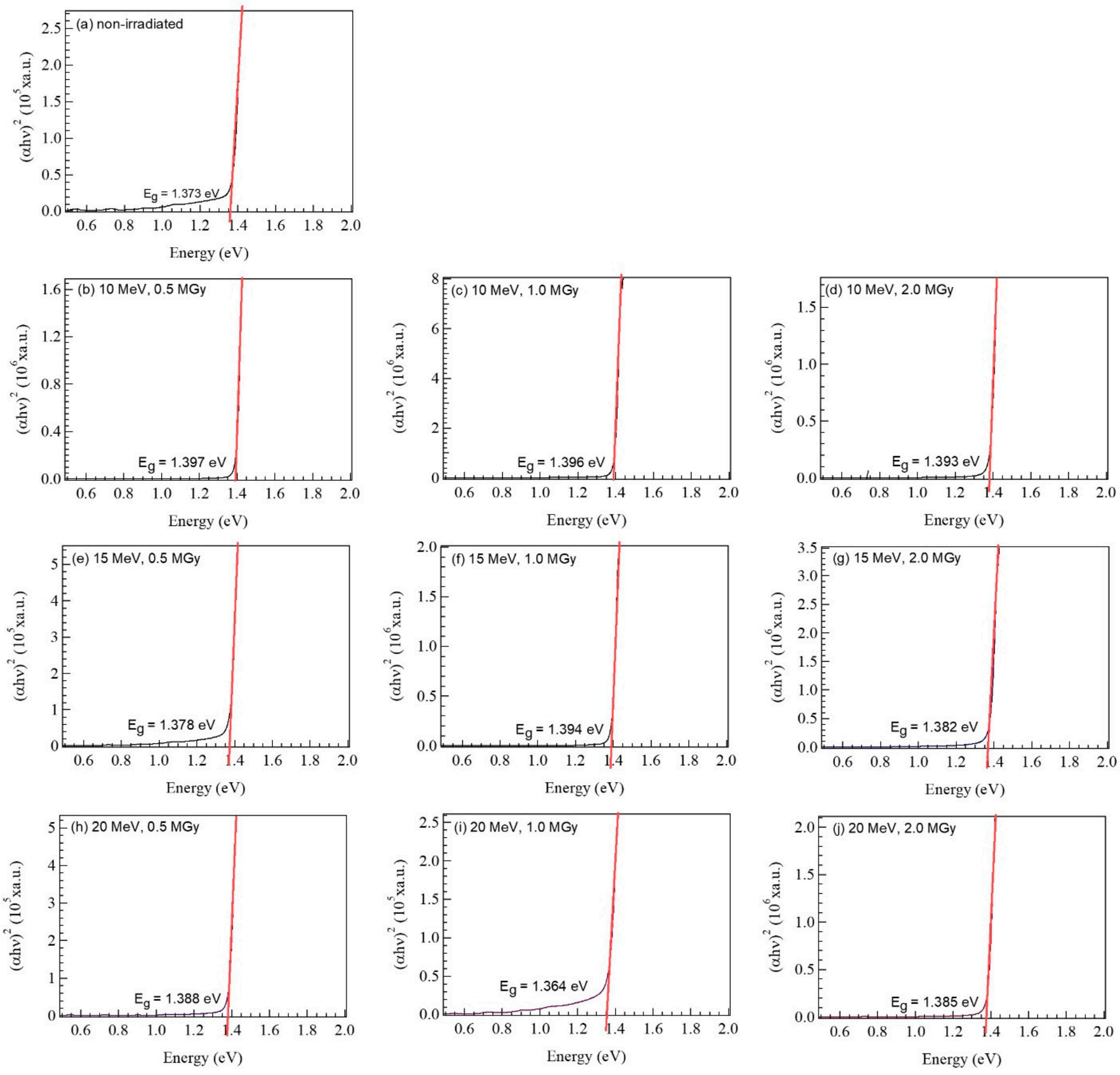

Figure S1 Tauc plot of $(\alpha h\nu)^2$ vs. photon energy ($h\nu$) of (a) the non-irradiated and (b-j) the irradiated GaAs samples with electron energies of 10, 15, and 20 MeV at electron doses of 0.5, 1.0, and 2.0 MGy.

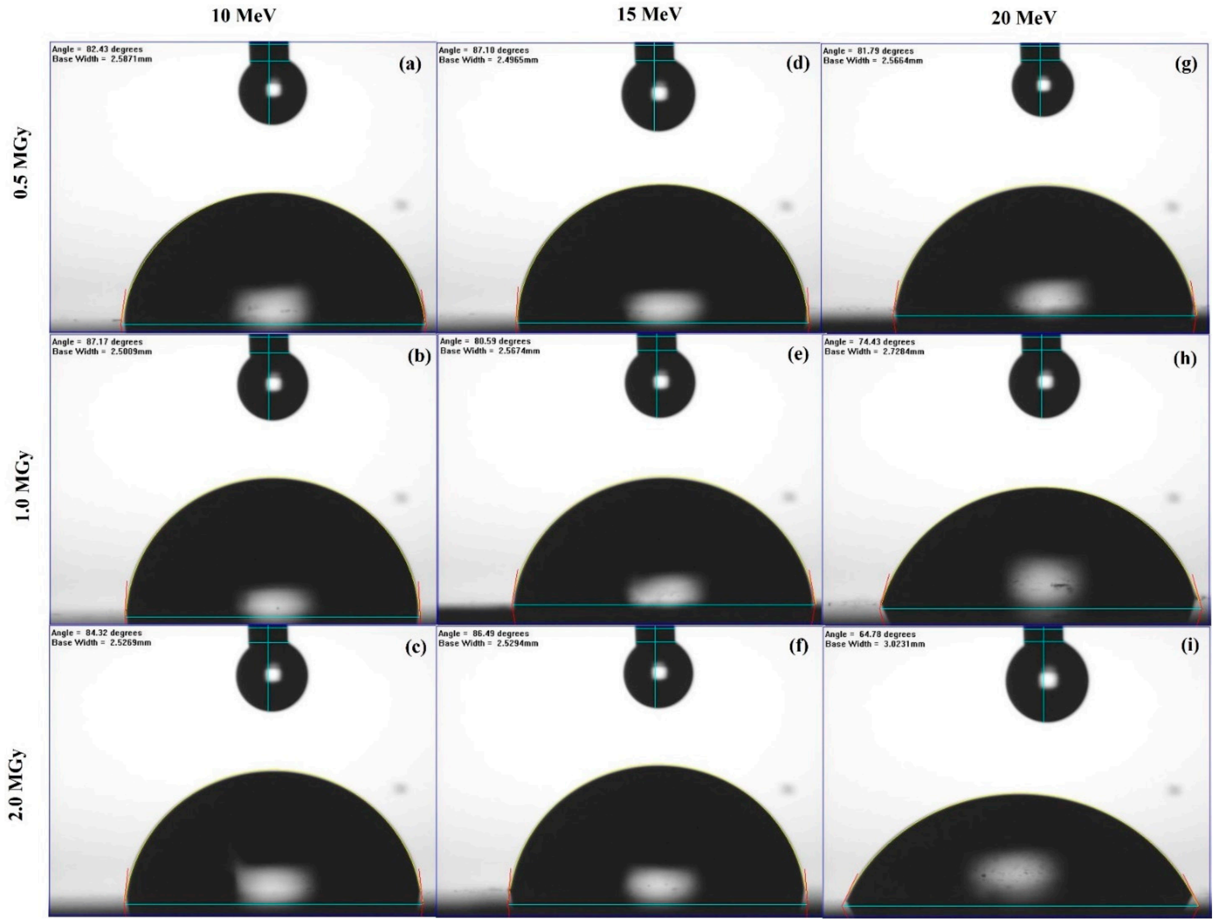

Figure S2 Contact angle measurement of the irradiated GaAs samples with electron energies of 10 (**a-c**), 15 (**d-f**), and 20 (**g-i**) MeV at electron doses of 0.5, 1.0, and 2.0 MGy, respectively.
